# Serendipitous In Situ Conservation of Faba Bean Landraces in Tunisia: A Case Study

**DOI:** 10.3390/genes11020236

**Published:** 2020-02-24

**Authors:** Elyes Babay, Khalil Khamassi, Wilma Sabetta, Monica Marilena Miazzi, Cinzia Montemurro, Domenico Pignone, Donatella Danzi, Mariella Matilde Finetti-Sialer, Giacomo Mangini

**Affiliations:** 1 National Gene Bank of Tunisia (BNG), Street Yesser Arafet, Charguia 1, Tunis 1080, Tunisia; e.babay@yahoo.fr; 2 Institut National de la Recherche Agronomique de Tunisie (INRAT), Agricultural Applied Biotechnology Laboratory (LR16INRAT06), University of Carthage, Rue Hédi Karray, Menzah 1, Tunis PC 1004, Tunisia; 3 Institut National de la Recherche Agronomique de Tunisie (INRAT), Field Crop Laboratory (LR16INRAT02), University of Carthage, Rue Hédi Karray, Menzah 1, Tunis PC 1004, Tunisia; khalilkhamassi@iresa.agrinet.tn; 4 Institute of Biosciences and Bioresources of the National Research Council (IBBR-CNR), Via Amendola 165/A, 70126 Bari, Italy; wilma.sabetta@ibbr.cnr.it (W.S.); domenico.pignone@ibbr.cnr.it (D.P.); donatella.danzi@ibbr.cnr.it (D.D.); 5 Department of Soil, Plant and Food Sciences (DiSSPA), Sect. Genetics and Plant Breeding, University of Bari Aldo Moro, Via Amendola 165/A, 70126 Bari, Italy; monicamarilena.miazzi@uniba.it (M.M.M.); cinzia.montemurro@uniba.it (C.M.)

**Keywords:** *Vicia faba* L., genetic diversity, SSR markers, in situ conservation

## Abstract

Cultivation of faba bean (*Vicia faba* L.) in Tunisia is largely based on improved varieties of the crop. However, a few farmers continue to produce local cultivars or landraces. The National Gene Bank of Tunisia (NGBT) recently launched a collection project for faba bean landraces, with special focus on the regions of the North West, traditionally devoted to cultivating grain legumes, and where around 80% of the total national faba bean cultivation area is located. The seed phenotypic features of the collected samples were studied, and the genetic diversity and population structure analyzed using simple sequence repeat markers. The genetic constitution of the present samples was compared to that of faba bean samples collected by teams of the International Center for Agricultural Research in the Dry Areas (ICARDA) in the 1970s in the same region, and stored at the ICARDA gene bank. The results of the diversity analysis demonstrate that the recently collected samples and those stored at ICARDA largely overlap, thus demonstrating that over the past 50 years, little genetic change has occurred to the local faba bean populations examined. These findings suggest that farmers serendipitously applied international best practices for in situ conservation of agricultural crops.

## 1. Introduction

Faba bean (*Vicia faba* L., 2n = 2x = 12) is a facultative cross-pollinating species with outcrossing rates varying between 1 and 55% depending on its environment; it belongs to the *Fabaceae* family, *Faboideae* subfamily, tribe of *Fabeae*, and is not interfertile with any other *Vicia* species [1]. The wild progenitor of *V. faba* is unknown, but recent archaeological excavations have allowed, in the Mont-Carmel (Mediterranean Levantine), the discovery of fossilized seeds that are compatible with a wild progenitor of this crop, dating as back as 14,000 ybp [2]. Considering other archaeological evidences, such as those relating to findings in Tel el Kerkh [3], northwest Syria, it is possible to hypothesize that this species has been domesticated since the Neolithic era, and that the wild progenitor, possibly distributed in small habitats, was entirely domesticated and then became extinct [2,3,4,5]. According to Cole [6] and Cubero [7], the spread of faba bean from its center of origin to other countries could have involved five routes. In the Mediterranean, in particular, faba bean mainly spread through two routes: the first across Anatolia to Greece, the Illyric coast (possibly the Danubian regions), and then to Italy; the second, beginning at the Nile Delta, moving towards the West, along the North African Mediterranean coast, to the Maghreb and then to the Iberian Peninsula. It is worth mentioning that, in this regard, North Africa and Tunisia in particular constitute a center of primary and secondary diversification of several agricultural and wild species [8].

In Tunisia, faba bean covers more than 70% (59,583 ha) of the total area annually devoted to grain legume crops [9]. Approximately half of the area is cultivated with grain legume types meant for fresh pod consumption; the rest as forage (plant and seeds) is mainly based on small seeded types. The average productivity in Tunisia is 1.03 t/ha, 40% below the world average [10]. This is mainly due to the parasitic weed broomrape (*Orobanche crenata* Forssk. and *O. foetida* Poir.) and drought stress occurring in faba bean-growing areas [11]. Until the last century, most crops consisted of landraces, often named after the farmer who selected them or after areas where they were grown [12]. Some landraces (‘Batata’, ‘Malti’, ‘Chemchali’ or ‘Masri local’) are still grown by farmers and seeds can be bought from local informal markets.

In recent years, thanks to significant achievements in crop breeding, modern high-yielding varieties are widely used in cultivation and have almost completely replaced local populations and landraces [11]. The increase in yield was obtained mainly with breeding programs targeted at tolerance to abiotic (heat and drought) and biotic (foliar diseases and parasitic weeds) stresses. Moreover, recent breeding efforts are directed towards the development of new cultivars with low anti-nutritional compounds (vicine and convicine), to improve the quality and utilization efficiency of faba in human diet and for livestock feed [13]. Different molecular tools were used to investigate genetic diversity in grain legume species [14,15,16,17]. Some attempts to evaluate genetic variability have also been made for Tunisian faba bean germplasm. The analysis of isozyme [18] and sequence specific amplified polymorphism (SSAP) [19] markers analyzed in nine Tunisian *Vicia faba* collections has indicated a certain degree of genetic cohesiveness. Using simple sequence repeat (SSR) markers, 16 faba bean accessions, selected from 42 populations collected across eight southern oases arid agro-ecosystems, were analyzed, evidencing genetic cohesiveness among the studied samples [19,20,21], together with a low level of variability among accessions. In both reports, the authors stated that intense seed exchange among farmers had led to a leveled degree of genetic diversity among those populations.

In the 1960s, major concerns focused on the genetic erosion of biodiversity, eventually leading to fostering of ex situ conservation efforts and the creation of gene banks [22]. As a result, in the latest decades of the 20th century, several different research centers organized collection missions for crop diversity in order to secure the local germplasm before it was completely lost [23]. Within the frame of “emergency” collections, from the seventies until the early nineties, ICARDA, among others, carried on a series of faba bean collection missions in North Africa. During that period, several faba bean samples were retrieved from different regions, in particular from Tunisia. At the end of the 20th century, taking into account the evident loss of genetic diversity in the Mediterranean [24], specific attention was paid to the practice of in situ conservation of crops. This is the conservation of agricultural genetic resources on farms located in the same areas where local communities had developed them, with specific attention to neglected crops [25,26]. According to Duc et al. [23], in situ conservation of biodiversity may contribute to the development of the best-adapted materials for local agronomic practices and involve farmers in the selection process through participatory breeding [27]. Within this frame, NGBT started an ongoing program of genetic resource collection in different areas of Tunisia. The aim was to preserve Tunisian crop gene-pools from genetic erosion and characterize local germplasm, thanks to an integrated approach, including on-farm conservation of local germplasm and landraces.

In order to better plan an in situ conservation strategy for faba bean and to understand the possible loss of genetic diversity in Tunisian *Vicia faba* germplasm, the genetic structure of the samples collected in recent years was compared with those collected by ICARDA in the 1970s. This paper reports on the results of this comparison.

## 2. Materials and Methods

### 2.1. Plant Material

The plant material used in the present study consisted of a collection of 51 Tunisian local faba bean samples (Table 1). It included 29 samples collected during 2016–2018 by the NGBT and 22 faba bean accessions collected by ICARDA, starting from the seventies until the early nineties. The NGBT samples were collected in the governorates of Beja and Jendouba (Figure S1) characterized by annual average rainfall of 800 and 600 mm, respectively. The passport data and ethno-botanical information of the NGBT samples are available at NGBT. The samples of ICARDA (labelled ICAR) were derived from collection missions conducted from the seventies until the early nineties in North Tunisia (Beja, Bizerte, and Siliana).

### 2.2. Seed Phenotypic Traits

Five seeds of each sample were randomly selected to determine average seed size. The three axial dimensions of seed length (L), width (W), and thickness (T) were measured using a Vernier caliper (Gilson Tools, Japan) with accuracy of 0.05 mm. The geometric mean diameter (Dg) was calculated by using the equation reported by Mohsenin [28]:

Dg = (L × W × T)^1/3^ (1)

The sphericity (φ) of faba bean seeds was calculated using the following formula:

φ = [(L × W × T)^1/3^/L]*100 (2)

### 2.3. DNA Extraction and SSR Assays

Total genomic DNA was extracted from fresh young leaves—five plants per sample—using the cetyltrimethyl ammonium bromide (CTAB) method, as described by Fulton et al. [29]. DNA concentration was determined using a NanoDrop^TM^ ND-2000 (Thermo Scientific, MA, USA) and the quality was verified by separation on 0.8% agarose gel. Equal DNA quantities of the five plants of the sample were then pooled, and all DNA samples were diluted to a standard working concentration of 50 ng/µl by adding ultrapure water (Gibco, Invitrogen, USA).

A set of 11 simple sequence repeats (SSRs) markers, retrieved from the literature [30,31,32] were used for this study (Table S1). A preliminary assay was carried out in order to evaluate the robustness of PCR reaction and the reproducibility of the fragments. In particular, different faba samples randomly chosen were analysed considering technical replicates. The PCR conditions for each SSR markers were set up at best conditions, considering an annealing temperature ranging from 45 to 60 °C. The fragments produced were separated on 2.0% agarose gel containing Nancy-520 DNA Gel Stain (Sigma Genosys, St. Louis, MO, USA), and visualized under UV light. The amplification reactions were performed in a final volume of 20 µl, containing the template DNA (50 ng), the 5′end of each forward primer with the M13 (21 bp) tail, the reverse primer (M13) labeled with fluorescent dye (FAM, VIC, PET, or NED). PCR reactions were performed in a thermal cycler (Bio-Rad Laboratories, Hercules, CA, USA) as follows: an initial denaturing step at 95 °C for 3 min, followed by 36 cycles of 94 °C for 20 s, 56 °C for 50 s, 72°C for 1 min, and a final extension step at 72 °C for 7 min. The amplification products were detected by automatic capillary sequencer ABI PRISM 3100 Genetic Analyzer (Applied Biosystems, Waltham, MA, USA), and the fragments were analyzed with GeneMapper genotyping software version 5.0 (Thermo Fisher Scientific, Waltham, MA, USA). The internal molecular weight standard was GeneScanTM 600 LIZ dye Size Standard (Thermo Fisher Scientific, Waltham, MA, USA).

### 2.4. Data Analysis

Hierarchical ascending classification (HAC) clustering analysis based on dissimilarity matrix of morphometric seed data (L, W, T, Dg, and φ) was performed to evaluate the relationship among the faba samples using XLSTAT statistical software ver. 2016.2 (Addinsoft Inc, New York, USA).

The genetic indices, number of different alleles (*Na*), Shannon’s information index (*I*), observed Heterozygosity (*Ho*), expected Heterozygosity (*He*), Fixation Index (*F*), and private alleles were calculated using GenAlEx version 6.5 [33]. The allelic data were used to obtain a similarity matrix, from which a dendrogram was constructed using the UPGMA algorithm with MEGA ver. 4 [34]. The molecular data were processed using STRUCTURE ver. 2.3.4 [35]. The number of sub-populations (K) was estimated by 10 independent runs for each K (from 1 to 10), applying the admixture model, 500000 Markov Chain Monte Carlo (MCMC) repetitions, and a 100000 burn-in period. The means of the log-likelihood estimates for each K were calculated. The true K was determined with the Evanno test [36] using STRUCTURE HARVESTER [37]. Analysis of molecular variance (AMOVA) was used to partition the genetic variation into inter- and intra-gene pool diversities in faba using GenAlEx program ver. 6.5, with 1000 permutations.

## 3. Results

### 3.1. Variation in Seeds Phenotypic Traits

**Figure 1:**
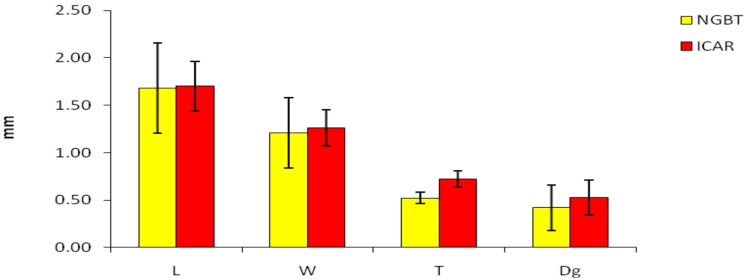
Length (L), width (W), thickness (T), and geometric mean diameter (Dg) averages of samples collected by the National Gene Bank of Tunisia (NGBT) and Agricultural Research in the Dry Areas (ICAR). Lines represent the standard deviation.

Morphometric seed traits (L, W, T, Dg, and φ) were measured in the samples belonging to both the NGBT and ICARDA faba bean collection. The average mean of the three principal axial dimensions (L, W, and T) and Dg of the NGBT and ICAR groups are shown in Figure 1. No statistically significant differences for these morphometric seed traits were detected between the two groups. The values of faba bean sphericity (φ) were calculated by using the geometric mean diameter and length data. No significant difference was found for these traits when comparing the NGBT and ICAR groups.

**Figure 2:**
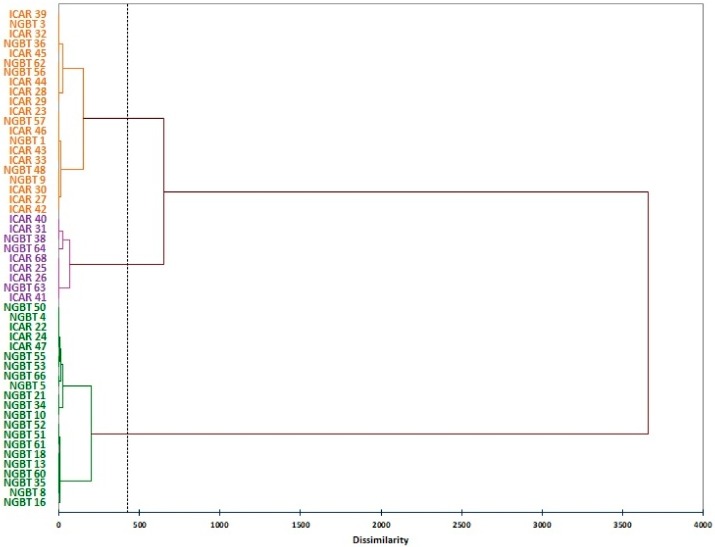
Dendrogram based on dissimilarity matrix calculated from morphometric seed traits in the faba bean collection split in samples collected by the National Gene Bank of Tunisia (NGBT) and Agricultural Research in the Dry Areas (ICAR). The colors orange, purple, and green were used to distinguish between the medium, large, and small seed type clusters, respectively.

The values of morphometric seed traits of each samples were used to calculate a dissimilarity matrix based on Euclidean distances. A dendrogram was obtained starting from the matrix, in which three main clusters are evidenced, corresponding to three seed types—large, medium (called also equina), and small (Figure 2). Each cluster included both NGBT and ICAR samples with no reference to the area of origin.

### 3.2. Molecular Variation of Faba Bean Collection

In order to evaluate the genetic diversity of 51 faba bean samples, a set of 11 SSR markers was used (Table 1 and Table S1). A total number of 94 alleles were identified, ranging from 3 (loci M22 and M46) to 22 (locus VFG41), with an average of 10.2 alleles per locus (Table S2).

AMOVA did not show a molecular diversity among the 29 NGBT faba beans when these were grouped according to the collection sites (El Hamra, Oued Ghrib and Fouazia), suggesting that the NGBT faba bean samples belonged to one genetically cohesive population (Table S3).

According to these results, we investigated the genetic diversity among samples collected in recent years by NGBT and ICAR during the 1970s, in the same Tunisian areas. Similar values for the number of different alleles, Shannon’s information index, Heterozygosity expected, Heterozygosity observed, Diversity index, and Fixation index were found between the two groups, although the ICAR samples appeared slightly more fixed (Table 2).

AMOVA indicated that the NGBT and ICAR groups have no statistical difference, at the molecular level (Table 3), as most of the diversity clearly appeared within groups, with only a limited variation occurring among them.

**Figure 3:**
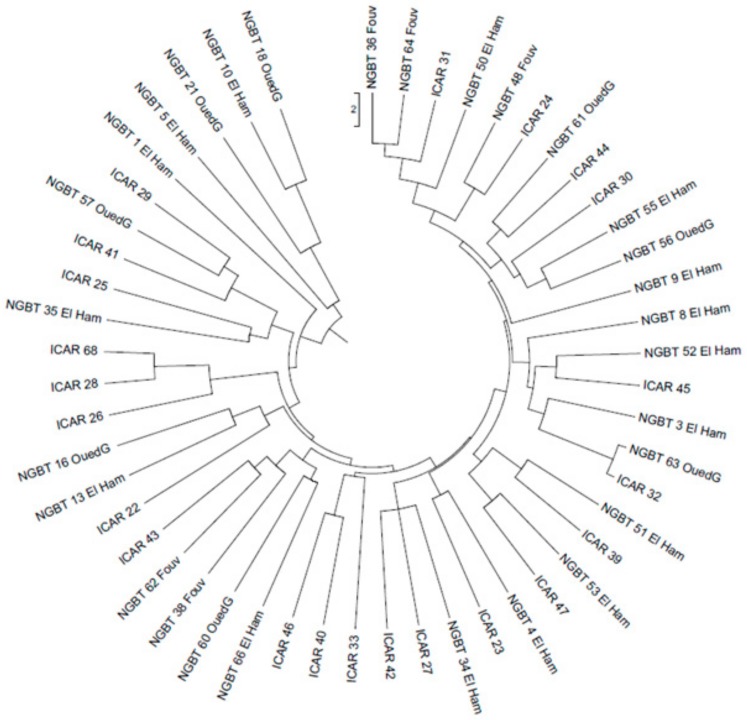
Dendrogram of faba bean collection split in samples collected by the National Gene Bank of Tunisia (NGBT) and Agricultural Research in the Dry Areas (ICAR) resulting from the UPGMA cluster analysis based on similarity matrix obtained from 11 SSR allelic data.

**Figure 4:**
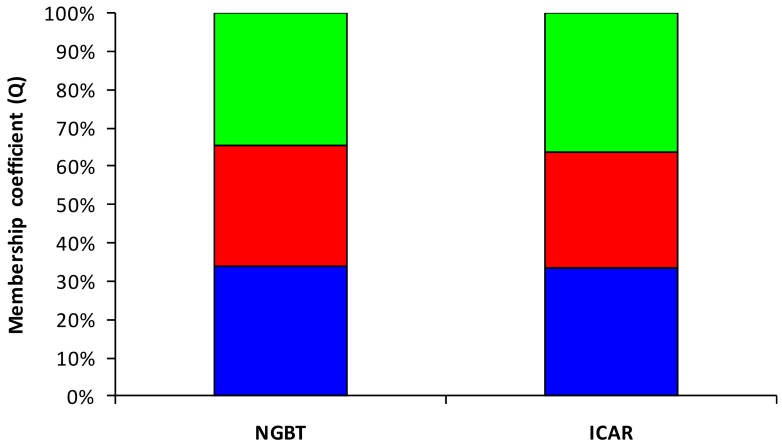
Membership coefficient (Q) mean of the faba bean collection split in samples collected by the National Gene Bank of Tunisia (NGBT) and Agricultural Research in the Dry Areas (ICARDA). The different colors indicate the three subpopulations detected using a Bayesian approach (blue: subpopulation 1; red: subpopulation 2, and green: subpopulation 3).

To define the genetic relationships among the faba bean samples, the similarity matrix obtained was also used to produce a UPGMA dendrogram (Figure 3). The clustering showed that the NGBT samples were admixed with ICAR ones, thus suggesting that the two groups could be considered a unique meta-population. Total faba bean collection was also evaluated with Bayesian clustering modeling, performed using SSR allelic data generated according to 11 SSR markers. As the clustering model presumes the underlying existence of K clusters, an Evanno test [36] was performed that yielded K=3 as the highest log-likelihood (Figure S2). Nevertheless, each sample analyzed showed to belong to all three clusters identified, and none of them predominantly pertained to a specific cluster (Figure 4). These results seemingly indicate that the Tunisian faba collection was structured in three subpopulations that do not correspond to NGTB and ICAR samples’ distinction.

## 4. Discussion

Faba bean is an important crop for sustainable agriculture in marginal areas and advanced agricultural systems, as it plays an important role in soil fertility and nitrogen fixation, being able to grow in diverse climatic and soil conditions. Although faba bean is less consumed in western countries as human food, it is considered one of the main sources of cheap protein and energy for many people in Africa, parts of Asia, and Latin America, where many people cannot afford to buy meat [38]. Seed size and shape are characters of polygenic control [39,40] and have undergone strong selection measures by farmers during evolution of the crop. Seed size is considered a key trait in the study of the historical evolution of this crop based on archaeological remains and findings [2]. This selection pressure can still be found in the habits of farmers, who manually select seeds to be sown in the next season [41], a habit that was followed in many Mediterranean regions until modern times and lost only on introduction of improved varieties. This manual selection procedure resulted in the formation of peculiar landraces, such as “Larga di Leonforte” [42]. However, this study noted that Tunisian farmers did not practice seed selection, and have not done so for the last 50 years.

The analysis of morphometric seed traits and molecular markers carried out in this work did not distinguish patterns in the distribution of morphological and genetic variations. All the samples collected by the NGBT teams appeared to belong to a single, genetically cohesive population. Similar results were observed for the faba bean samples obtained by ICARDA and collected from the same areas. When all faba samples were analyzed together, there were no differences between the NGBT and ICARDA groups. In fact, our molecular analyses and seed morphometric variation study demonstrated that the two groups belonged to a single meta population.

Seed size is one of the most important morphological traits responsible for yield, and a major target for breeding. Several studies have led to the mapping of QTLs/genes for seed weight/size in soybean [43], chickpea [44], and lentil [45]. In faba bean, the genetic control of these traits is still unclear, although consensus linkage maps have been produced [43,46].

Our data confirmed that morphometric seed traits were not associated to the markers used. This implies that any selection of lines based on seed traits still retains a variable level of genetic diversity potentially associated to other traits, such as adaptation or resistance, an issue to be taken into account in any faba bean-breeding program based on phenotypic data.

The absence of genetic differentiation in different collection sites might depend on many factors. The absence of human selection pressure does not force crop adaptation in a specific direction. The outbreeding habit of faba bean is a second factor; in fact, while the advanced breeding varieties favor inbreeding in search of higher stability, as requested by UPOV standards, the landraces are generally quite allogamous [47,48]. Recent studies have also demonstrated that the level of allogamy might depend on the species of pollinating insects [49]. A further, effective mechanism could be derived by the spontaneous seed exchange practice occurring among farmers. In informal seed markets, seed exchange by farmers favors the establishment of a landrace in a given environment with uniform agro-climatic characteristics. The farmers cultivating these landraces are actually the relics of a once wider cultivation area. All these factors act synergically to produce the observed genetic differentiation patterns.

The Evanno test yielded three subpopulations within the faba bean collection. In addition, each sample had a coefficient membership lower than 0.50, thus denying that it predominantly belonged to one of these subpopulations. This further supports the fact that the two meta-populations, NGBT and ICARDA, are subsamples of a unique population collected in the same area at different times.

The biological significance of the three subpopulations detected with STRUCTURE is unclear, and might depend on the presence of genetic signals that do not parallel clear-cut characteristics. Nevertheless, several authors have reported that hierarchical analyses, such as those based on STRUCTURE or similar software, relay on strict assumptions that might not completely apply to the case studies, thus resulting in incorrect evaluation of the population structure [50].

In conclusion, unintended conservation of ancient faba bean germplasm in Tunisian farms is witnessed, because the farmers cultivating faba bean landraces do not follow seed selection, as well as owing to concurring factors such as the high level of cross-pollination, and the consistent presence of pollinators (due to the limited use or absence of insecticides). This beneficial situation is an empirical application of best practices recommended by research institutions for on-farm conservation of plant genetic resources. In their quest to feed their families and their precious animals, the Tunisian farmers in the small villages mentioned above serendipitously stored and protected their faba bean genetic resources. These findings and implications should be further discussed in the broadest context possible. Future research directions should also be highlighted.

## Figures and Tables

**Table 1:** List of faba bean samples included in the study.

Id Name	Local Name	Governorate	Location	Longitude (E)	Latitude (N)	Seed Type
NGBT 1	Malti	Beja	El Hamra	9.011641	36.52219	Large
NGBT 3	Malti	″	″	9.011641	36.52219	Large
NGBT 4	Malti	″	″	9.011641	36.52219	Large
NGBT 5	Chemchali	″	″	9.011641	36.52219	Small
NGBT 8	Chemchali	″	″	9.011641	36.52219	Small
NGBT 9		″	″	9.012502	36.52136	Medium
NGBT 10	-	″	″	9.012502	36.52136	Small
NGBT 13	-	″	″	9.012502	36.52136	Small
NGBT 34	-	″	″	9.012502	36.52136	Small
NGBT 35	-	″	″	9.012502	36.52136	Small
NGBT 50	Chemchali	″	″	9.011641	36.52219	Small
NGBT 51	Chemchali	″	″	9.011641	36.52219	Small
NGBT 52	Chemchali	″	″	9.011641	36.52219	Small
NGBT 53	Chemchali	″	″	9.011641	36.52219	Small
NGBT 55	-	″	″	9.012502	36.52136	Medium
NGBT 66	-	″	″	9.012502	36.52136	Medium
NGBT 16	-	Jendouba	Oued Ghrib	8.412815	36.37286	Small
NGBT 18	-	″	″	8.412815	36.37286	Large
NGBT 21	Bachar	″	″	8.414203	36.37533	Small
NGBT 56	-	″	″	8.414203	36.37533	Large
NGBT 57	-	″	″	8.414203	36.37533	Large
NGBT 60	-	″	″	8.412815	36.37286	Small
NGBT 61	-	″	″	8.412815	36.37286	Large
NGBT 63	-	″	″	8.414203	36.37533	Large
NGBT 36	Malti	″	Fouazia	8.404811	36.40103	Large
NGBT 38	Malti	″	″	8.404811	36.40103	Large
NGBT 48	Malti	″	″	8.404811	36.40103	Large
NGBT 62	Malti	″	″	8.404811	36.40103	Large
NGBT 64	Malti	″	″	8.404811	36.40103	Large
ICAR 22	Local	n.a.	n.a.	n.a.	n.a.	Small
ICAR 23	Seville	n.a.	n.a.	n.a.	n.a.	Large
ICAR 24	Misri 32	Siliana	n.a.	9.616670	36.35000	Small
ICAR 25	Local	n.a.	n.a.	n.a.	n.a.	Large
ICAR 26	-	Bizerte	n.a.	n.a.	n.a.	Large
ICAR 27	-	Beja	n.a.	n.a.	n.a.	Medium
ICAR 28	-	″	n.a.	n.a.	n.a.	Large
ICAR 29	-	n.a.	n.a.	n.a.	n.a.	Large
ICAR 30	Local	Bizerte	n.a.	n.a.	n.a.	Small
ICAR 31	Local	n.a.	n.a.	n.a.	n.a.	Large
ICAR 32	Local	n.a.	n.a.	n.a.	n.a.	Small
ICAR 33	-	n.a.	n.a.	n.a.	n.a.	Small
ICAR 39	Local	Bizerte	n.a.	n.a.	n.a.	Medium
ICAR 40	Malti 24	″	n.a.	9.666670	37.05000	Medium
ICAR 41	Malti 25	″	n.a.	9.666680	37.06000	Small
ICAR 42	Misri 39	Beja	n.a.	9.583330	36.66670	Small
ICAR 43	Misri 41	″	n.a.	9.216670	36.71670	Small
ICAR 44	-	″	n.a.	n.a.	n.a.	Medium
ICAR 45	-	″	n.a.	n.a.	n.a.	Small
ICAR 46	Local	Bizerte	n.a.	n.a.	n.a.	Large
ICAR 47	Local	n.a.	n.a.	n.a.	n.a.	Small
ICAR 68	Local	n.a.	n.a.	n.a.	n.a.	Large

n.a. data not available.

**Table 2:** Number of different alleles (*Na*), Shannon’s information index (*I*), Heterozygosity observed (*Ho*), Heterozygosity expected (*He*), Fixation Index (*F*), and private alleles of faba bean collection split in samples recently collected by the National Gene Bank of Tunisia (NGBT) and Agricultural Research in the Dry Areas (ICARDA).

	N° Samples	Na	I	Ho	He	F	Private Alleles
NGBT	29	7	1.428	0.621	0.658	0.027	23
ICARDA	22	6	1.392	0.546	0.657	0.145	12
Whole Collection	51	7	1.410	0.584	0.657	0.086	

**Table 3:** Analysis of molecular variance (AMOVA) of faba bean collection split in samples collected by the National Gene Bank of Tunisia (NGBT) and Agricultural Research in the Dry Areas (ICARDA).

Source of Variation	df	SS	MS	Est. Var.	*p*-Values
Among groups	1	3.650	3.65	0.01	n.s
Within groups	49	161.94	3.31	3.3	
Total	50	165.59		3.31	

df = degree of freedom, SS = sum of squares, MS = mean squares, Est. Var. = estimate of variance, *p*-value.

## Supplementary Materials

The following are available online at https://www.mdpi.com/2073-4425/11/2/236/s1, **Figure S1:** NGBT sampling **Figure S2:** Delta K for differing numbers of subpopulations (K) estimated for faba bean collection with 11 markers; **Table S1:** List of SSR markers used for genetic analysis of faba bean collection; **Table S2:** Number of different alleles (*Na*), Shannon’s information index (*I*), Heterozygosity observed (*Ho*), Heterozygosity expected (*He*), and Fixation Index (*F*) of 11 SSR markers used in faba bean collection; **Table S3:** Analysis of molecular variance (AMOVA) of 29 NGTB faba bean split according to collection sites (El Hamra, Oued Ghrib, and Fouazia).
