# Supplementary material for: Serendipitous In Situ Conservation of Faba Bean Landraces in Tunisia: A Case Study
Source: Genes (Basel). 2020 Feb 24;11(2):236. doi: 10.3390/genes11020236 (PMC7074078; doi:10.3390/genes11020236)
Supplement: Supplementary file 1 [file genes-11-00236-s001.zip › Supplementary_file genes-713699_proofreading/Figure S2.pdf]

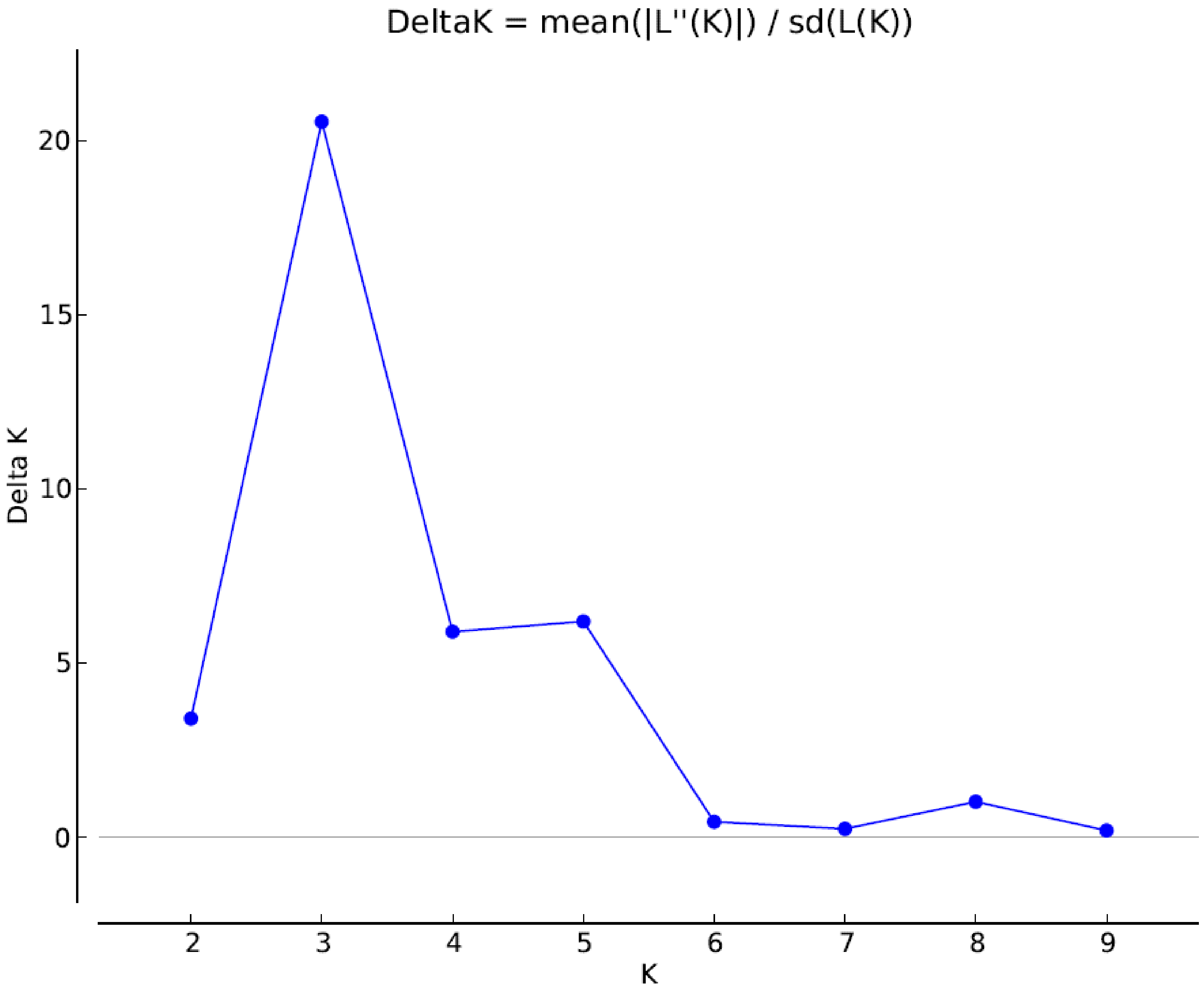

Figure S2. Delta K for differing numbers of subpopulations (K) estimated in faba bean collection with 11 markers.
