# Supplementary material for: Serendipitous In Situ Conservation of Faba Bean Landraces in Tunisia: A Case Study
Source: Genes (Basel). 2020 Feb 24;11(2):236. doi: 10.3390/genes11020236 (PMC7074078; doi:10.3390/genes11020236)
Supplement: Supplementary file 1 [file genes-11-00236-s001.zip › Supplementary_file genes-713699_proofreading/Table S1.pdf]

Table S1. List of SSR markers used for genetic analysis of faba bean collection.

SSR Locus	Sequence (5'-3')	Ta (°C)	Repeat motif	References
M9	F: AATCACAAGCGACGACGAC	58	(GA)8	[30]
	R: GCGGAATATGCAGACCAAAT			
M22	F: TCGCAATAGCACAGAACCTG	58	(CCACCG)3	"
	R: GATCAAACTCCCCAACCTCA			
M25	F: CGTCGTGAAAATCATGGAGA	57	(ATC)6	"
	R: CATTATTATTACACCCGCCTCA			
M27	F: TCCGATCAATTCCCTGAGAC	57	(CGAAAA)3	"
	R: CGGTATCTTGCTTTCCTTCG			
M36	F: GCGGGTTTATTCCATC	47	(CCA)8	"
	R: TCCGTTTGCGTAGC			
M41	F: CAACGCGGCAGTTAAAGAAT	57	(GGAACC)3	"
	R: CAGGTATGGCTGACACCTCA			
M43	F: TTCTGATGAGAAGCCAACCA	57	(AAT)5	"
	R: GGGGGAGGAACCAGATTATT			
M46	F: GGATGGATTGATTCTCCAACA	57	(ATAA)4	"
	R: GCATAACTAACACATTATGCAGGA			
SSR1	F: ACCAGGCAGAGTTAGATAAGCA	56	(GGAACC)3	[31]
	R: GTCACTCGGATGTCCAGGTAT			
VFG1	F: TTTCAGCAAACTAGAACCAATC	53	(AG)15	[32]
	R: GGCATTCAGTTTTTACCTTGTA			
VFG41	F: AGCCCATGGTTCAAATGCAA	53	$(AG)_7$ + 10	"
	R: GCAGTCATGCCACTGCTTA			
