# Supplementary material for: Serendipitous In Situ Conservation of Faba Bean Landraces in Tunisia: A Case Study
Source: Genes (Basel). 2020 Feb 24;11(2):236. doi: 10.3390/genes11020236 (PMC7074078; doi:10.3390/genes11020236)
Supplement: Supplementary file 1 [file genes-11-00236-s001.zip › Supplementary_file genes-713699_proofreading/Table S2.pdf]

Table S2. Number of different alleles (*Na*), Shannon's information index (*I*), Heterozygosity observed (*Ho*), Heterozygosity expected (*He*) and Fixation Index (*F*) of 11 SSR markers used in faba bean collection.

	M9	M22	M25	M27	M36	M41	M43	M46	SSR1	VFG1	VFG41
Na	6	3	8	8	9	5	6	3	10	14	22
I	1.411	0.447	1.368	1.278	1.709	1.347	1.304	0.764	1.808	2.216	2.646
Ho	0.447	0.250	0.733	0.45	0.897	0.640	0.408	0.600	0.940	0.848	0.237
He	0.674	0.225	0.662	0.603	0.779	0.707	0.66	0.473	0.792	0.866	0.890
F	0.337	-0.113	-0.107	0.254	-0.151	0.094	0.381	-0.269	-0.187	0.021	0.734
