# Supplementary material for: Serendipitous In Situ Conservation of Faba Bean Landraces in Tunisia: A Case Study
Source: Genes (Basel). 2020 Feb 24;11(2):236. doi: 10.3390/genes11020236 (PMC7074078; doi:10.3390/genes11020236)
Supplement: Supplementary file 1 [file genes-11-00236-s001.zip › Supplementary_file genes-713699_proofreading/Table S3.pdf]

Table S3. Analysis of molecular variance (AMOVA) of 29 NGTB faba bean split according to the collecting sites (El Hamra, Oued Ghrib and Fouazia).

**Source of variation**	**df**	**SS**	**MS**	**Est. Var.**	***p* values**
**Among groups**	1	3.65	3.65	0.01	n.s
**Within groups**	49	161.94	3.31	3.3	
**Total**	50	165.59		3.31	

df = degree of freedom; SS = sum of squares; MS = mean squares; Est. var. = estimate of variance.
